# Phaeochromocytoma-induced secondary takotsubo syndrome

**DOI:** 10.1093/ehjci/jead053

**Published:** 2023-04-04

**Authors:** Simon M Frey, Michael J Zellweger, Katharina Glatz, Maurice Pradella, Philip Haaf

**Affiliations:** Department of Cardiology, University Hospital Basel, University of Basel, Petersgraben 4, Basel CH-4031, Switzerland; Department of Cardiology, University Hospital Basel, University of Basel, Petersgraben 4, Basel CH-4031, Switzerland; Pathology, Institute of Medical Genetics and Pathology, University Hospital Basel, University of Basel, Basel, Switzerland; Department of Radiology, Clinic of Radiology and Nuclear Medicine, University Hospital Basel, University of Basel, Switzerland; Department of Cardiology, University Hospital Basel, University of Basel, Petersgraben 4, Basel CH-4031, Switzerland

An otherwise healthy 41-year-old female patient presented with an onset of cardiogenic shock (blood pressure 97/79 mmHg, heart rate 160/min). Electrocardiogram revealed sinus tachycardia without evidence of ischaemia (see Supplementary data online, *Figure S1*). She reported a history of palpitations, chest pain, hot flashes, and feeling more short-tempered lately. Due to significantly elevated high-sensitivity cardiac troponin T (3614 ng/L) and N-terminal prohormone of brain natriuretic peptide (7208 ng/L), coronary artery disease was ruled out by invasive coronary angiography (see Supplementary data online, *Video S1*). Echocardiography showed a severely impaired left ventricular ejection fraction (LVEF 15%) with apical akinesia and hypercontractility of the basal segments (see Supplementary data online, *Video S2*). Cardiovascular magnetic resonance imaging confirmed the severely impaired ejection fraction (LVEF 29%) and regional wall motion abnormalities (*Panel A* + *B*, Supplementary data online, *Video S3*). T2 mapping revealed myocardial oedema in the apical two-thirds of the myocardium with corresponding mildly elevated myocardial extracellular volume (*C*). There was no myocardial infarction or focal fibrosis on late gadolinium enhancement images (*D*). Free serum metanephrine, normetanephrine, and chromogranin were significantly elevated. A dedicated abdominal magnetic resonance imaging demonstrated a round, well-demarcated tumour of the right adrenal gland (38 × 45 × 46 mm) which corroborated the diagnosis of an adrenal phaeochromocytoma with secondary takotsubo syndrome. Alpha and beta adrenergic blockade were administered prior to laparoscopic right adrenalectomy, and a 50 × 40 × 35 mm measuring tumour was removed (*F* + *G*). Histopathology confirmed the diagnosis of a phaeochromocytoma with small nests of basophilic neuroendocrine cells expressing chromogranin A (*H*). In a follow-up echocardiography 2 weeks post-surgery, LVEF and wall motion abnormalities had completely recovered (see Supplementary data online, *Video S4*). This case illustrates the rare (reversible) condition of a secondary takotsubo syndrome due to a phaeochromocytoma-induced catecholamine storm.

**Figure:**
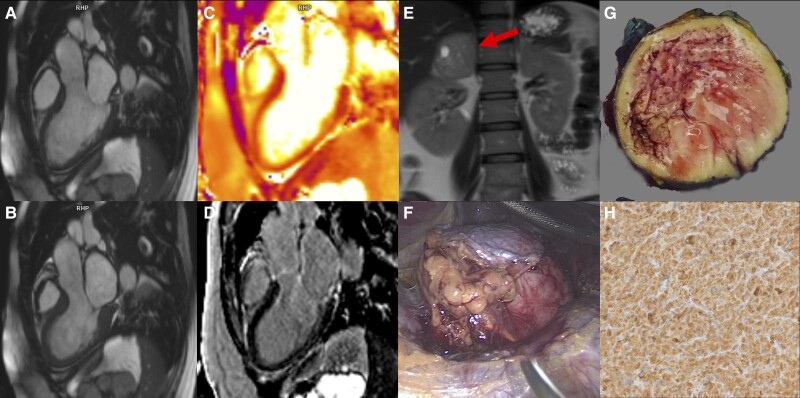


Supplementary data are available at *European Heart Journal - Cardiovascular Imaging* online.

**Consent**: The patient consented to the use of her anonymized health care data for publication as a case report.

**Data availability:** The data underlying this article will be shared on reasonable request to the corresponding author.

## Supplementary Material

jead053_Supplementary_Data

