# Supplementary figures and images for: Phaeochromocytoma-induced secondary takotsubo syndrome
Source: Eur Heart J Cardiovasc Imaging. 2023 Apr 4;24(6):e105. doi: 10.1093/ehjci/jead053 (PMC10243999; doi:10.1093/ehjci/jead053)

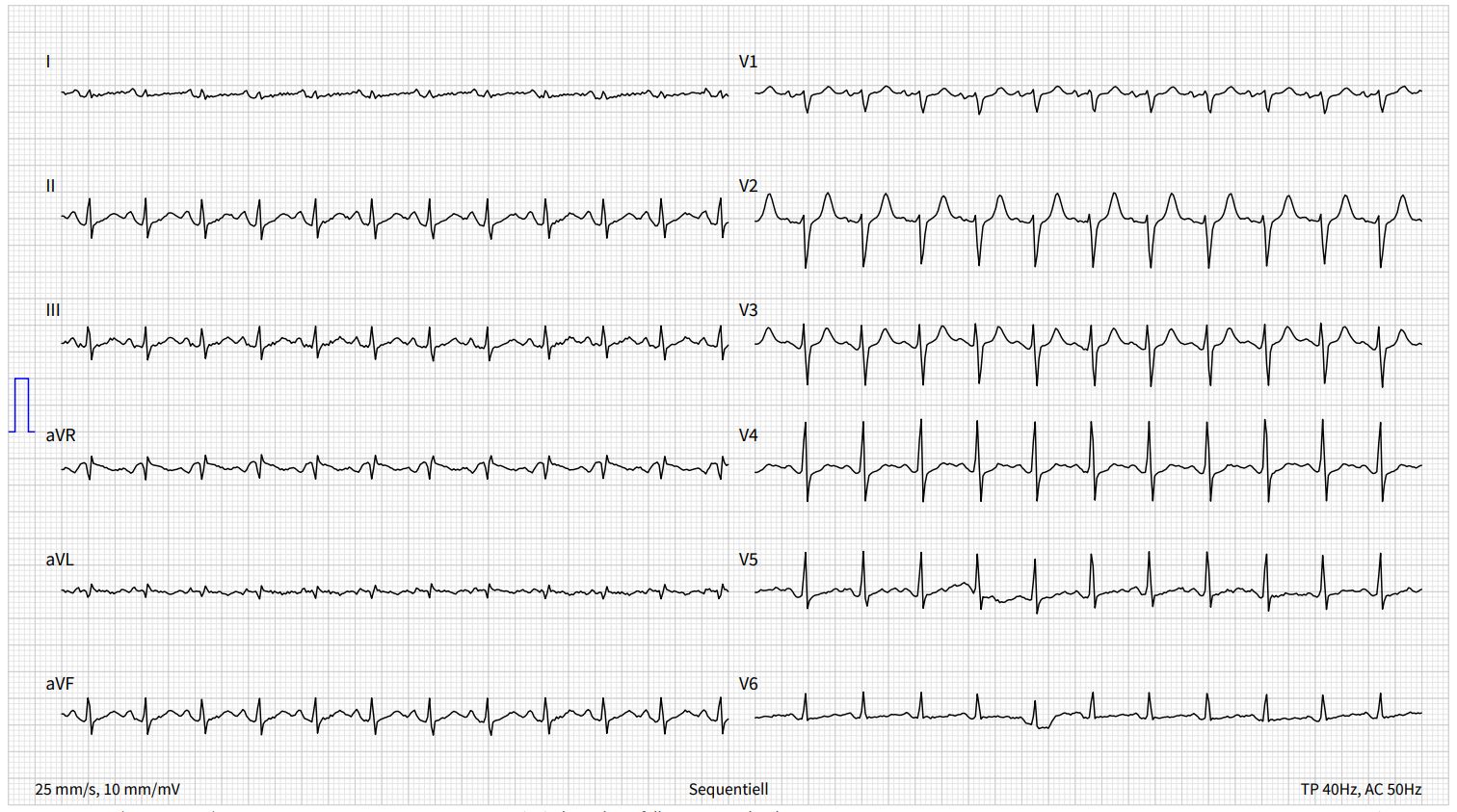

Supplement: jead053_Supplementary_Data [file jead053_supplementary_data.zip › Supplemental figure 1 (ECG).JPG]
